# A Peculiar Case

**Published:** 1872-12

**Authors:** Eugene Smith

**Affiliations:** Detroit


### A PECULIAR CASE.

By Eugene Smith, Detroit.

July 23d, I was consulted by Mrs. J. concerning her baby's right eye, the lids of which were very much swollen, as were also the temple and the scalp of the same side. The child was three weeks old, apparently quite healthy and strong, and had shown no indications of ill-health until three days previous. There was no abrasion of the skin nor had there been any. The right side of the face and head were of an erysipelatous hue.

Upon close examination I discovered four pimples and two holes about the size of a pin-head; the holes were in the outer surface of the lower lid, and the pimples on the temple and scalp; pus was oozing from one of the holes, while in the other three was evidently a *moving something*, which, on being extracted, with the forceps, proved to be a *grub* or *maggot* about one line in thickness, and nearly $\frac{1}{4}$ of an inch in length. The holes were perpendicular to the surface and about $\frac{1}{2}$ an inch in depth. The mother remarked that from the other hole a worm nearly as large had worked itself out.

Examining the other four papules I detected *motion* beneath the surface, and pinching off the very thin top of each, I extracted *four more*, of varying size, the first, however, being much the largest.

The worms resembled, in appearance, those we find in fruit.

Now the question is, what are they, and where did they come from? Aitkin, in his practice, vol. 1, p. 887, speaks of a "Bulama Boil," caused by the larvæ of an insect, and his discription of the worm answers very closely to those I removed, but in my case there was no regular boil.

Had there been an abrasion or wound, or conjunctival secretion, I would have considered them to be larvæ of the fly, but there was not; neither had there been anything of the kind; and I do not believe the ovæ could have been deposited in a sebaceous duct. The baby was as neat and clean, and as well taken care of, as possible.

I am at a loss to know how to account for their presence, and several physicians of many years' experience, to whom I showed the pathological specimens (?), had neither seen nor heard of a similar case.

That the oʌæ were deposited by an insect seems like the only rational way of accounting for their presence, but *how* is not clear to my mind. I should be pleased to have the opinion of the profession on the subject.

The child recovered after the removal of the worms. *Mich. University Med. Journal.*

